# Effects of dapagliflozin on heart rate variability, cardiac function, and short-term prognosis in early-onset post-myocardial infarction heart failure

**DOI:** 10.3389/fcvm.2024.1490316

**Published:** 2025-01-06

**Authors:** Le Zhou, Mingyuan Niu, Wei Chen, Qian Hu, Yi Chen, Xiaohong Geng, Jiani Gu

**Affiliations:** ^1^ Department of Cardiology, Shibei Hospital of Jing'an District, Shanghai, China; ^2^ Department of Cardiology, Shigatse People’s Hospital, Xizang, China; ^3^ Department of Cardiology, Zhabei Central Hospital of Jing’an District, Shanghai, China

**Keywords:** dapagliflozin, acute myocardial infarction, heart rate variability, soluble growth stimulation expressed gene 2 protein, N-terminal pro B-type natriuretic peptide

## Abstract

**Objective:**

To investigate the effects of dapagliflozin, in addition to standard therapy, on heart rate variability (HRV), soluble growth stimulation expressed gene 2 protein (sST2), N-terminal pro B-type natriuretic peptide (NT-proBNP), and echocardiographic parameters in patients with early-onset post-myocardial infarction heart failure (HF).

**Methods:**

A total of 98 patients with early-onset post-myocardial infarction HF were enrolled and randomly divided into a control group (*n* = 48, receiving standard therapy) and an observation group (*n* = 50, receiving standard therapy plus dapagliflozin 10 mg daily). HRV, cardiac function, and echocardiographic parameters were measured at baseline and after 24 weeks of treatment. Short-term prognosis and adverse events were also monitored.

**Results:**

Compared with the control group, the observation group showed significantly greater improvements in SDNN and SDANN (*P* < 0.05). Significant improvements were also observed in sST2 and NT-proBNP levels in the observation group compared to the control group (*P* < 0.05). Additionally, echocardiographic parameters, including EF, LVESD, LVEDD, IVST, LVMI, and E/e’, showed greater improvement in the observation group (*P* < 0.05). The incidence of major adverse cardiovascular events was lower in the observation group (*P* < 0.05). Multivariate logistic regression model revealed that dapagliflozin use was independently associated with a reduced risk of MACE (OR = 0.265, 95% CI: 0.097–0.724, *P* = 0.010).

**Conclusion:**

Early administration of dapagliflozin 10 mg, in addition to standard therapy, can improve autonomic function, cardiac function, and short-term prognosis in patients with early-onset post-myocardial infarction heart failure.

## 1 Introduction

Sodium-glucose cotransporter 2 inhibitors (SGLT2i) are a novel class of oral antidiabetic drugs that have garnered significant attention for their cardioprotective and renoprotective effects beyond glycemic control (1–3). Recent clinical studies have demonstrated that SGLT2i treatment can significantly reduce the risk of cardiovascular death and hospitalization due to worsening heart failure in patients with chronic heart failure across the full spectrum of left ventricular ejection fraction (LVEF), irrespective of the presence of diabetes (4, 5). The underlying mechanisms of these effects are complex and not yet fully understood, particularly regarding how SGLT2i influence the sympathetic and parasympathetic nervous function of the heart. Acute myocardial infarction (AMI) is one of the most common and significant causes of heart failure globally. Early-onset heart failure following AMI is defined as heart failure present at admission or occurring during hospitalization (6). The most frequent cause of early post-AMI sudden cardiac death is malignant arrhythmia (7). Enhanced sympathetic nervous activity and reduced parasympathetic activity are both indicative of the occurrence of malignant arrhythmias and poor clinical outcomes (8). Heart rate variability (HRV) is a key measure used to assess cardiac autonomic function.

Based on the above, this study investigates the effects of early initiation of dapagliflozin 10 mg (once daily, orally) on HRV, cardiac function, and echocardiographic parameters in patients with early-onset heart failure following AMI, regardless of the presence of diabetes, all of whom received standardized interventional and pharmacological treatment. The study also follows up on short-term prognostic indicators, with results reported as follows.

## 2 Materials and methods

### 2.1 Study subjects

This study included patients with early-onset heart failure following AMI admitted to the Cardiac Care Unit (CCU) of Shanghai Jing'an District Shibei Hospital between July 2021 and July 2023, regardless of whether they had concomitant diabetes. A total of 120 patients who met the inclusion and exclusion criteria were enrolled, with 98 completing follow-up.

The inclusion and exclusion criteria for this study are as follows. Inclusion Criteria: Based on the “Fourth Universal Definition of Myocardial Infarction” and the “2020 Expert Consensus on the Prevention and Treatment of Heart Failure after Myocardial Infarction,” patients were eligible if they had AMI with symptoms or signs of heart failure at admission or during hospitalization, and if their NT-proBNP levels were >125 pg/ml and sST2 levels were >35 ng/ml (4). Exclusion Criteria: (1) Severe renal insufficiency (eGFR ≤ 30 ml/min/1.73 m^2^) or severe hepatic insufficiency. (2) Uncorrectable cardiogenic shock, uncorrectable diabetic ketoacidosis, or recurrent genitourinary infections within the past six months. (3) Type 1 diabetes; severe valvular disease, cardiomyopathy, congenital heart disease, hematologic or immune system diseases, or malignant tumors. (4) Incomplete clinical data or inability to complete follow-up. (5) Prior use of SGLT2 inhibitors.

### 2.2 Methods

This study was approved by the Ethics Committee of Shanghai Jing'an District Shibei Hospital (Approval No. YL-20230424-16). All enrolled patients and their families provided written informed consent.

In this study, participants were randomly assigned to either the control group (48 patients) or the observation group (50 patients) using a computer-generated random number table. This randomization process was performed by an independent researcher who was not involved in patient recruitment or treatment administration. This study was designed as a single-blind study, where the outcome assessors, including those performing echocardiographic evaluations and symptom monitoring, were blinded to the treatment allocation.

Both groups of patients received standardized interventional and pharmacological treatment according to clinical guidelines and consensus. In addition to this standard treatment, the observation group received dapagliflozin tablets (10 mg, brand name: Farxiga, manufactured by AstraZeneca Pharmaceuticals Ltd., Approval No. National Drug Standard J20170040) within 24 h of enrollment, administered once daily in the morning, with no restrictions regarding food intake. The treatment duration was 24 months.

### 2.3 Observational indicators and follow-up

#### 2.3.1 Heart rate variability (HRV)

HRV was assessed in both groups using 24 h ambulatory electrocardiography. Data were collected within one week of enrollment and again at 24 weeks of follow-up. To minimize variability, HRV measurements were standardized by ensuring that recordings were performed at the same time of day for each patient (between 8:00 AM and 10:00 AM) and under a consistent resting state. Patients were instructed to rest quietly for at least 10 min before measurement to reduce potential interference from physical activity or stress. All HRV data were analyzed using the Lepu TH1222C02306C Holter monitor.

#### 2.3.2 Laboratory tests

Fasting venous blood samples (10 ml) were collected from both groups on the second day of enrollment and at 24 weeks of follow-up. Blood samples were analyzed using the AFIAS device (Badi Tech, South Korea) for sST2. Serum was separated by centrifugation, and NT-proBNP levels were measured using the Pylon Iris automatic immunoassay analyzer.

#### 2.3.3 Echocardiographic parameters

Echocardiography was performed within 24 h of enrollment and again at 24 weeks of follow-up. The Philips CX50 echocardiography system was used to measure the following parameters: ejection fraction (EF), left ventricular end-systolic dimension (LVESD, mm), left ventricular end-diastolic dimension (LVEDD, mm), interventricular septal thickness (IVST, mm), left ventricular mass index (LVMI, g·m^2^), and the E/e’ ratio.

All echocardiographic measurements were conducted by a single, experienced cardiologist who was trained in echocardiography. To ensure consistency, intra-observer variability was assessed by repeating the measurements for a subset of subjects at two different time points (enrollment and follow-up). The measurements were recorded by the same observer, and any discrepancies were resolved by a second review by the cardiologist. Additionally, all measurements were performed in a blinded manner, with the evaluator unaware of the patients’ clinical status and treatment group. A random sample of the echocardiographic recordings was further reviewed by a second independent cardiologist to evaluate inter-observer agreement.

#### 2.3.4 Major adverse cardiovascular events (MACE)

In this study, MACE were defined as the following events: (1) Rehospitalization due to worsening heart failure: Patients were considered to have experienced a MACE if they were rehospitalized due to acute exacerbation of heart failure, as determined by clinical symptoms (e.g., dyspnea, edema, fatigue) and confirmed by objective evidence (e.g., increased natriuretic peptides, echocardiographic findings). (2) Non-fatal acute coronary syndrome (ACS): Non-fatal ACS events included both non-fatal myocardial infarction and unstable angina. Diagnosis was confirmed through clinical presentation, biomarkers (e.g., troponin), and imaging results (e.g., coronary angiography). (3) Malignant arrhythmia: This category included clinically significant arrhythmias, such as ventricular tachycardia or fibrillation, requiring medical intervention or resulting in hospitalization. (4) Stroke: Stroke was diagnosed based on clinical presentation and confirmed by neuroimaging (CT or MRI), classified as ischemic or hemorrhagic.

All MACE events were independently adjudicated by a blinded endpoint committee, which reviewed patient medical records, including clinical notes, hospitalization records, laboratory results, and imaging studies. Any discrepancies in event classification were resolved through consensus.

In the analysis, MACE events were recorded and classified according to the aforementioned criteria. The safety and efficacy of dapagliflozin in heart failure management were assessed by the incidence of MACE, with further analysis of the impact of these events on the overall clinical outcomes.

#### 2.3.5 Follow-up

During the study, patients were followed to monitor medication adherence, any drug allergies, and to evaluate the efficacy and adverse effects of the medication (e.g., hypoglycemia, hypotension, urinary and reproductive system infections, ketoacidosis, fractures, lower limb amputations, bladder cancer). MACE were recorded, including recurrent acute coronary syndrome, malignant arrhythmias, hospitalization due to worsening heart failure, ischemic stroke, cardiovascular death, or all-cause mortality.

At the one-year follow-up, survival outcomes were assessed for both groups through telephone follow-up, which was conducted for all patients. Survival status was recorded, and rehospitalization rates were evaluated based on patient-reported data.

### 2.4 Statistical analysis

Statistical analyses were conducted using SPSS software version 26.0. Quantitative data are presented as means ± standard deviations (SD), and comparisons between groups were made using the *t*-test. Categorical data are presented as *n* (%), and the chi-square test was used for comparisons. A multivariate logistic regression analysis was conducted to identify independent risk factors for MACE in early-onset post-myocardial infarction heart failure patients. The dependent variable was the occurrence of MACE, and the independent variables included age, sex, comorbidities (hypertension, diabetes), and treatment regimen (dapagliflozin vs. standard therapy). The multivariate model was adjusted for potential confounders, and odds ratios (OR) with 95% confidence intervals (CI) were calculated. A *P*-value of <0.05 was considered statistically significant.

## 3 Results

### 3.1 Comparison of general characteristics

In the control group (*n* = 48), the average age was 68.05 ± 9.79 years; there were 28 men and 20 women. The smoking rate was 33.33%. The average body mass index (BMI) was 23.66 ± 2.57 kg/m^2^. The baseline heart rate was 82.19 ± 22.56 beats per minute. The comorbid conditions included hypertension in 38 patients, diabetes in 30, hyperlipidemia in 6, stroke in 18, a history of percutaneous coronary intervention (PCI) in 8, and a history of coronary artery bypass grafting (CABG) in 4. The average door-to-balloon time was 77.92 ± 18.16 min. Average systolic blood pressure was 111.88 ± 14.79 mmHg, and average diastolic blood pressure was 75.40 ± 9.51 mmHg. The proportion of anterior wall myocardial infarctions was 33.33%, and the proportion of patients with multi-vessel coronary artery disease was 41.67%. The average number of stents implanted was 1.12 ± 0.93. The average GRACE score was 158.42 ± 39.77, and the average Gensini score was 57.09 ± 34.16. Upon discharge, medication use included antiplatelet agents (97.92%), β-blockers (91.67%), statins (95.83%), nitrates (37.5%), angiotensin receptor-neurolysin inhibitors (ARNI) (83.33%), mineralocorticoid receptor antagonists (MRA) (41.67%), loop diuretics (31.25%), biguanides (52.08%), GLP-1 receptor agonists (4.17%), and DPP-4 inhibitors (20.83%).

In the observation group (*n* = 50), the average age was 65.27 ± 11.87 years; there were 30 men and 20 women. The smoking rate was 24%. The average BMI was 23.50 ± 2.70 kg/m^2^. The baseline heart rate was 79.76 ± 18.65 beats per minute. The comorbid conditions included hypertension in 37 patients, diabetes in 25, hyperlipidemia in 8, stroke in 14, a history of PCI in 10, and a history of CABG in 4. The average door-to-balloon time was 79.13 ± 16.99 min. Average systolic blood pressure was 110.25 ± 26.07 mmHg, and average diastolic blood pressure was 75.83 ± 9.62 mmHg. The proportion of anterior wall myocardial infarctions was 36%, and the proportion of patients with multi-vessel coronary artery disease was 32%. The average number of stents implanted was 1.04 ± 0.55. The average GRACE score was 145.40 ± 32.17, and the average Gensini score was 52.44 ± 32.39. Upon discharge, medication use included antiplatelet agents (98%), β-blockers (90%), statins (98%), nitrates (30%), angiotensin receptor-neurolysin inhibitors (ARNI) (72%), mineralocorticoid receptor antagonists (MRA) (44%), loop diuretics (34%), biguanides (40%), GLP-1 receptor agonists (4%), and DPP-4 inhibitors (24%). There were no statistically significant differences in any of the characteristics between the two groups (*P* > 0.05), indicating comparability, as shown in Table 1.

**Table 1:** Comparison of general data between the two groups (mean ± SD) or *M* (*P_25_, P_75_*).

Parameter	Control group (*n* = 48)	Observation group (*n* = 50)	*χ^2^/t*	*P*
Age (years)	68.05 ± 9.79	65.27 ± 11.87	1.288	0.201
Gender				
Male (%)	28 (58.33%)	30 (60%)	0.028	0.867
Female (%)	20 (41.67%)	20 (40%)	0.028	0.867
Smoking (%)	16 (33.33%)	12 (24%)	1.045	0.307
Body mass index (kg/m^2^)	23.66 ± 2.57	23.50 ± 2.70	0.297	0.767
Comorbidities				
Hypertension (%)	38 (79.17%)	37 (74%)	0.364	0.546
Diabetes mellitus (%)	30 (62.5%)	25 (50%)	1.554	0.213
Hyperlipidemia (%)	6 (12.5%)	8 (16%)	0.245	0.621
Stroke (%)	18 (37.5%)	14 (28%)	1.005	0.316
History of percutaneous coronary intervention (PCI) (%)	8 (16.67%)	10 (20%)	0.181	0.670
History of coronary artery bypass grafting (CABG) (%)	4 (8.33%)	4 (8%)	0.000	1.000
Duration of D2W (min)	77.92 ± 18.16	79.13 ± 16.99	−0.240	0.812
Heart rate (beats·min^−1^)	82.19 ± 22.56	79.76 ± 18.65	0.567	0.572
Systolic blood pressure (mmHg)	111.88 ± 14.79	110.25 ± 26.07	0.271	0.788
Diastolic blood pressure (mmHg)	75.40 ± 9.51	75.83 ± 9.62	−0.159	0.875
Anterior wall myocardial infarction (%)	16 (33.33%)	18 (36%)	0.077	0.782
Multi-vessel coronary artery disease (%)	20 (41.67%)	16 (32%)	0.985	0.321
Number of stents implanted	1.12 ± 0.927	1.04 ± 0.55	0.358	0.722
GRACE score	158.42 ± 39.77	145.40 ± 32.17	1.685	0.096
Gensini score	57.09 ± 34.16	52.44 ± 32.39	0.654	0.515
Baseline laboratory indicators				
Scr (umol·L^−1^)	77.33 ± 21.09	67.19 ± 15.56	2.888	0.510
UA (umol·L^−1^)	358.51 ± 111.14	352.67 ± 98.74	0.293	0.770
GLU (mmol·L^−1^)	6.83 ± 2.35	7.34 ± 3.05	−0.812	0.419
HbA1c (%)	6.52 ± 1.35	7.10 ± 1.66	−1.901	0.060
TC (mmol·L^−1^)	4.73 ± 0.82	4.61 ± 0.98	0.717	0.475
TG (mmol·L^−1^)	1.59 ± 0.68	1.74 ± 1.15	−0.840	0.403
HDL-C (mmol·L^−1^)	1.02 ± 0.19	1.02 ± 0.24	0.091	0.928
LDL-C (mmol·L^−1^)	2.98 ± 0.66	2.84 ± 0.86	0.949	0.345
TnI (ng·ml^−1^)	74.95 ± 27.83	71.59 ± 33.50	0.310	0.759
Discharge medications (%)				
Aspirin	47 (97.92%)	49 (98%)	1.000	0.742
P2Y12 receptor inhibitors	47 (97.92%)	49 (98%)	1.000	0.742
β-blockers	44 (91.67%)	45 (90%)	0.000	1.000
Statins	46 (95.83%)	49 (98%)	0.001	0.971
Nitrates	18 (37.5%)	15 (30%)	0.617	0.432
ARNI	40 (83.33%)	36 (72%)	1.807	0.179
MRA	20 (41.67%)	22 (44%)	0.054	0.816
Loop diuretics	15 (31.25%)	17 (34%)	0.084	0.772
Biguanides	25 (52.08%)	20 (40%)	1.440	0.230
GLP-1 receptor agonists	2 (4.17%)	2 (4%)	0.000	1.000
DPP-4 inhibitors	10(20.83%)	12(24%)	0.141	0.707

Note: Continuous variables were compared using the *t*-test; categorical variables were compared using the chi-square test. ARNI, angiotensin receptor-neurolysin inhibitor; MRA, mineralocorticoid receptor antagonist.

### 3.2 Comparison of HRV, cardiac function, and echocardiographic parameters before and after follow-up between the two groups

Compared to the control group, the observation group showed more pronounced improvements in SDNN and SDANN, with significant statistical differences (*P* < 0.05). Additionally, the observation group demonstrated greater improvements in sST2 and NT-proBNP compared to the control group, with significant statistical differences (*P* < 0.05). The observation group also exhibited more notable improvements in EF, LVESD, LVEDD, IVST, LVMI, and E/e’ compared to the control group, with significant statistical differences (*P* < 0.05), as shown in Table 2.

**Table 2:** Comparison of HRV, cardiac function indexes, and echocardiographic parameters between the two groups after 24 weeks (mean ± SD).

Parameter	Control group (*n* = 48)				Observation group (*n* = 50)				Between-group comparison	
	Baseline	24 weeks	*t*	*P*	Baseline	24 weeks	*t*	*P*	*t*	*P*
HRV										
SDNN (ms)	81.08 ± 14.86	93.80 ± 23.86	−2.386	0.028	92.51 ± 33.63	130.30 ± 35.60	−2.556	0.023	−3.633	0.001
SDANN (ms)	66.28 ± 17.34	83.50 ± 29.44	−2.217	0.039	73.91 ± 32.00	116.30 ± 38.34	−2.881	0.012	−2.867	0.007
rMSSD (ms)	42.36 ± 13.79	66.87 ± 34.81	−3.767	0.001	52.81 ± 23.83	85.88 ± 27.73	−3.770	0.001	−1.910	0.064
PNN50 (%)	5.04 ± 2.31	7.31 ± 1.97	−3.055	0.007	6.37 ± 2.95	8.14 ± 1.05	−2.163	0.048	−1.478	0.149
VLF (ms^2^)	1,513.70 ± 872.56	2,274.92 ± 778.28	−2.932	0.009	1,868.81 ± 977.33	2,711.15 ± 676.31	−2.870	0.012	−1.734	0.092
LF (ms^2^)	1,239.48 ± 805.85	1,627.42 ± 746.41	−1.328	0.200	1,226.95 ± 510.02	1,949.86 ± 515.71	−3.805	0.002	−1.510	0.141
HF (ms^2^)	735.91 ± 315.41	1,024.76 ± 451.39	−2.133	0.046	581.82 ± 237.99	854.16 ± 320.38	−2.967	0.010	1.245	0.222
Cardiac function										
sST2 (ng·ml^−1^)	51.58 ± 29.37	23.75 ± 8.73	3.578	0.003	57.56 ± 33.42	14.29 ± 8.30	5.331	0.000	3.130	0.004
NT-proBNP (pg·ml^−1^)	1,184.71 ± 652.27	949.41 ± 488.73	2.212	0.046	1,599.67 ± 936.19	436.40 ± 236.34	3.854	0.002	3.114	0.004
Echocardiographic parameters										
EF value (%)	48.33 ± 10.22	55.28 ± 7.53	−2.911	0.010	49.00 ± 9.37	60.94 ± 6.89	−5.014	0.000	−2.318	0.027
LVESD (mm)	34.50 ± 3.60	31.28 ± 4.08	2.836	0.011	36.88 ± 4.26	27.71 ± 2.85	8.562	0.000	2.985	0.005
LVEDD (mm)	49.12 ± 4.86	44.00 ± 2.98	3.695	0.002	50.35 ± 4.96	40.13 ± 3.72	5.358	0.000	3.312	0.002
IVST (mm)	10.47 ± 1.18	9.94 ± 0.90	2.496	0.024	10.41 ± 1.18	9.29 ± 0.85	3.271	0.005	2.157	0.039
LVMI (g·m^−2^)	131.10 ± 16.48	124.21 ± 15.25	4.098	0.001	130.62 ± 17.79	109.23 ± 16.01	4.259	0.001	2.792	0.009
E/e^’^	9.71 ± 1.16	9.06 ± 0.75	2.281	0.037	9.59 ± 1.33	8.53 ± 0.72	3.816	0.002	2.107	0.043

Note: The *t*-test was used for comparing continuous data.

### 3.3 Comparison of adverse event incidence rates

The incidence of adverse events in the control group was 8.33%, including 2 cases of urinary tract infections, 2 cases of hypotension, and a total of 4 adverse events. The incidence of adverse events in the observation group was 8%, including 1 case of urinary tract infection, 3 cases of hypotension, and a total of 4 adverse events. The difference between the control and observation groups was not statistically significant (*P* > 0.05), indicating that dapagliflozin has a high safety profile (see Table 3).

**Table 3:** Comparison of adverse events between the two groups.

Group	Urinary tract infection	Hypotension	Hypoglycemia	Non-ketotic hyperglycemic DKA	Total incidence (%)
Control (*n* = 48)	2	2	0	0	4 (8.33)
Observation (*n* = 50)	1	3	0	0	4 (8.00)*

* Compared to the control group: *P > 0.05.

### 3.4 Comparison of MACE events

**Figure 1:**
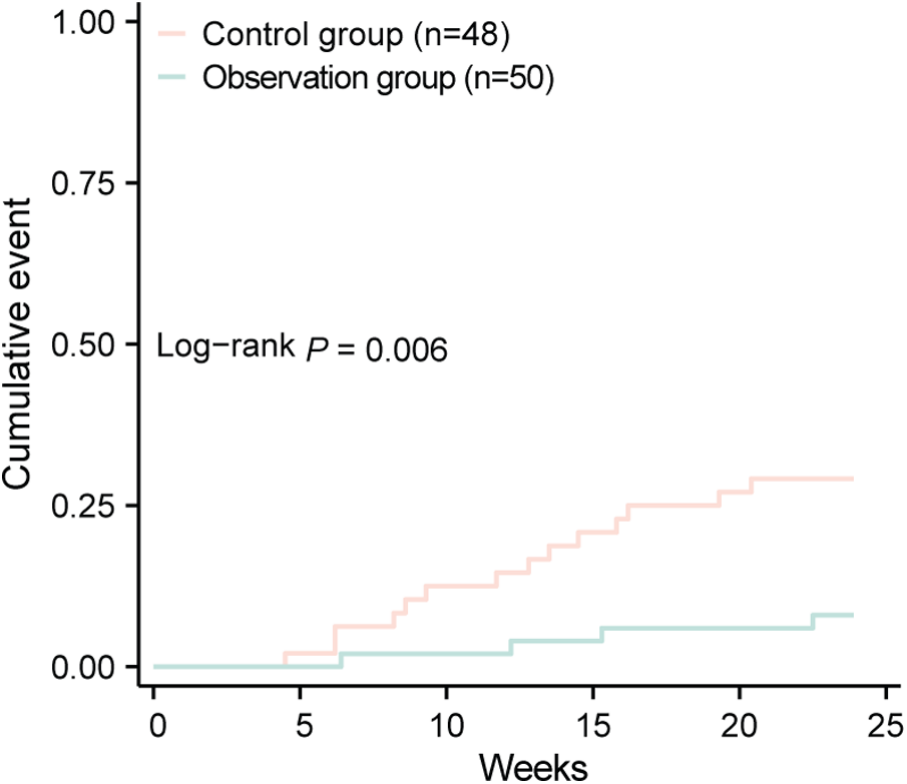
Comparison of cumulative events between two groups.

The incidence of MACE in the control group was 29.17%, including 7 cases of rehospitalization due to worsening heart function, 4 cases of non-fatal acute coronary syndrome, 2 cases of malignant arrhythmia, and 1 case of stroke, totaling 14 MACE events. In the observation group, the incidence of MACE was 8.00%, including 2 cases of rehospitalization due to worsening heart function and 2 cases of non-fatal acute coronary syndrome, totaling 4 MACE events. The incidence of MACE in the observation group was significantly lower than in the control group (*P* < 0.05), indicating that dapagliflozin improves the short-term prognosis of heart failure following early myocardial infarction (see Table 4; Figure 1). A logistic regression analysis was performed to identify the risk factors associated with the occurrence of MACE in early-onset post-myocardial infarction heart failure patients (see Table 5). The analysis included baseline variables such as age, comorbidities (hypertension, diabetes), and treatment regimen (dapagliflozin vs. standard therapy). In the univariate analysis, several variables were found to be associated with MACE, including hyperlipidemia and dapagliflozin use. The multivariate logistic regression model, adjusted for these confounders, revealed that dapagliflozin use was independently associated with a reduced risk of MACE (OR = 0.265, 95% CI: 0.097–0.724, *P* = 0.010). Notably, the use of dapagliflozin was associated with a 73.5% reduction in the odds of experiencing MACE, independent of other clinical variables, suggesting its potential protective effect in this cohort of patients (see Table 5).

**Table 4:** Comparison of MACE events between the two groups.

Group	Rehospitalization due to worsening heart function	Non-fatal ACS	Malignant arrhythmia	Stroke	Total incidence (%)
Control (*n* = 48)	7	4	2	1	14 (29.17)
Observation (*n* = 50)	2	2	0	0	4 (8.00)*

*P < 0.05.

**Table 5:** Logistic regression analysis of risk factors for MACE events in early-onset post-myocardial infarction heart failure.

Clinical Characteristics	β	S.E	*χ* ^2^	OR	95% CI	*P*-value	β (adjusted)	S.E (adjusted)	*χ*^2^ (adjusted)	OR (adjusted)	95% CI (adjusted)	*P*-value (adjusted)
Age (years)	0.116	0.143	0.658	1.123	0.849–1.486	0.417						
Gender (male vs. female)	−0.232	0.164	2.001	0.793	0.575–1.094	0.157						
Smoking	0.114	0.159	0.514	1.121	0.821–1.531	0.473						
Body Mass Index (kg/m^2^)	0.201	0.177	1.290	1.223	0.864–1.730	0.256						
Hypertension	−0.063	0.154	0.167	0.939	0.694–1.270	0.682						
Diabetes mellitus	0.084	0.136	0.381	1.088	0.833–1.420	0.537						
Hyperlipidemia	1.780	0.894	3.964	5.930	1.028–34.200	0.046	1.355	0.948	2.043	3.877	0.605–24.856	0.153
Dapagliflozin (yes vs. not)	−1.623	0.371	19.138	0.197	0.095–0.408	<0.001	−1.327	0.512	6.717	0.265	0.097–0.724	0.010

Note: Adjusted OR and 95% CI are calculated after adjusting for variables with *P* < 0.05 in univariate logistic regression.

At the one-year follow-up, survival outcomes were assessed for both groups. In the control group (*n* = 48), 3 patients (6.25%) died during the follow-up period, whereas no deaths occurred in the observation group (*n* = 50). Regarding rehospitalization rates, the observation group had five patients, significantly lower than the 19 patients in the control group (*χ*^2^ = 11.590, *P* < 0.001).

## 4 Discussion

After AMI, various electrophysiological disturbances, such as ventricular arrhythmias and atrial fibrillation, commonly occur. These disturbances are associated with increased sympathetic nervous system activity post-AMI, leading to activation of the neuroendocrine system. This, along with inflammatory and immune responses, contributes to a series of myocardial and electrical remodeling processes. HRV is used to assess sympathetic and parasympathetic nervous function. Abnormal HRV indicates autonomic dysfunction, with parameters including time-domain indices (SDNN, SDANN, rMSSD, PNN50) and frequency-domain indices (VLF, LF, HF). Reduced HRV suggests increased sympathetic activity and/or decreased parasympathetic activity, which are strong and independent predictors of mortality in AMI patients. Our study found that early use of dapagliflozin in post-AMI heart failure significantly increased SDNN and SDANN, thereby improving cardiac autonomic function. This finding is consistent with the results from the EMBODY study (9). It is hypothesized that the underlying mechanisms may include: SGLT2i reducing volume load and lowering blood pressure without inducing compensatory heart rate increases. This clinical phenomenon suggests that SGLT2i may suppress sympathetic nervous activity. Further research indicates that SGLT2i may improve insulin resistance by lowering leptin and glucose levels, ameliorate anemia, reduce activation of carotid bodies, and inhibit neuronal activation in the third ventricle of the hypothalamus by lowering blood sodium levels, thus exerting a sympatholytic effect (10). Additionally, Nguyen et al. (11) observed in animal studies that SGLT2i might influence cardiovascular activity through multiple neural nuclei, including the hypothalamic paraventricular nucleus, endopiriform nucleus, and periaqueductal gray matter, thereby increasing parasympathetic activity and reducing blood pressure and heart rate. Moreover, our findings align with Nassif et al. (12), demonstrating dapagliflozin's benefits across a spectrum of heart failure phenotypes. Unlike Kosiborod et al., which emphasized patient-reported outcomes (KCCQ-CSS), our study focuses on early-onset post-myocardial infarction HF, highlighting improvements in biomarkers (sST2, NT-proBNP), autonomic function (HRV), echocardiographic parameters, and reduced MACE risk. These complementary results provide additional mechanistic insights into dapagliflozin's therapeutic effects.

Progressive ventricular remodeling following AMI is a significant contributor to heart failure. Therefore, early inhibition or delay of post-AMI ventricular remodeling is crucial for preventing the onset of heart failure. BNP has potent effects in counteracting myocardial hypertrophy and fibrosis. Elevated BNP levels can antagonize the activated sympathetic and renin-angiotensin-aldosterone systems, with higher serum BNP or NT-proBNP concentrations indicating an increased risk of myocardial remodeling and heart failure. ST2, a member of the interleukin-1 receptor family, is involved in the inflammatory response within the infarcted myocardial region and is associated with myocardial fibrosis and ventricular remodeling, affecting the prognosis of myocardial infarction (13). Elevated serum sST2 levels have been independently associated with increased mortality and other adverse outcomes (13). ST2 primarily originates from cardiac fibroblasts and myocytes and is relatively unaffected by age, gender, body mass index, or renal function. Animal studies have shown that dapagliflozin increases myocardial IL-10 levels, promotes the infiltration of M2 macrophages, inhibits myofibroblast differentiation, and reduces collagen synthesis, thereby exerting anti-fibrotic effects on the heart (14). Our study found that early use of dapagliflozin in post-AMI heart failure significantly reduced sST2 and NT-proBNP levels, suggesting that dapagliflozin may improve myocardial fibrosis and remodeling following early myocardial infarction.

Cardiac imaging plays a crucial role in the diagnosis and management of ventricular remodeling after AMI. Echocardiography is a convenient, rapid, and safe method for dynamically assessing cardiac structure and function, and is the preferred clinical approach for evaluating both systolic and diastolic cardiac function. Key indicators of systolic function include LVEF, LVEDV, and LVESV. Diastolic function is assessed using parameters such as the E/e’ ratio, the e’ velocity of the interventricular septum/left lateral wall, the maximum tricuspid regurgitant velocity, and the left atrial volume index. Our study found that in patients with early post-AMI heart failure, treatment with dapagliflozin significantly improved LVEF, with more notable increases compared to the control group. Additionally, LVESD, LVEDD, IVST, LVMI, and the E/e’ ratio were all significantly reduced, indicating that dapagliflozin can enhance both systolic and diastolic cardiac function in these patients.

In summary, dapagliflozin improves autonomic function, cardiac function, and short-term prognosis in patients with early post-AMI heart failure. This effect may be related to improvements in electrical and myocardial remodeling. However, the study is limited by its small sample size and short follow-up period. Future research with larger, high-quality, multicenter randomized controlled trials is needed to provide further evidence for improving the prognosis of AMI patients.

## Data Availability

The original contributions presented in the study are included in the article/Supplementary Material, further inquiries can be directed to the corresponding author.
